# 0596. Plasma «drug» of cardiovascular dysfunction in the rat model of hemorrhagic shock

**DOI:** 10.1186/2197-425X-2-S1-P39

**Published:** 2014-09-26

**Authors:** N Sennoun, M Toussaint-Hacquard, A Lecomte, L Chevreux, T Lecompte, S Gross, B Levy

**Affiliations:** French Blood Establishment-Lorraine Champagne, Vandoeuvre-lès-Nancy, France; University of Sciences and Technology Houari-Boumediene, Cellular and Molecular Biology, Alger, Algeria; Shock Research Group - Avenir Inserm U 961, Vandoeuvre-lès-Nancy, France

## Introduction

Hemorrhagic shock (HS) often cause coagulopathy and cardiovascular dysfunction. The extent of these alterations correlates with the severity of HS. Fresh Frozen Plasma use is usually designed to restore coagulation proteins levels. Clinical and experimental studies have shown that resuscitation with fresh plasma is associated with improved outcome after severe hemorrhagic shock.

## Objectives

The benefical effect of FFP on coagulopathy is well documented but its effect on vascular dysfunction is not clear. We hypothesized that in addition to its effects on hemostasis, FFP has other protective effect on cardiovascular dysfunction.

## Methods

A Wistar rat model of Hemorrhagic shock (HS) was used. Rats were anesthetized and instrumented for continuous monitoring of blood pressure, heart rate and body temperature.

Rats with cannulated jugular and femoral veins were subjected to withdrawals of 20mL/kg body weight of blood for 15min; resuscitation fluids (vol/vol blood loss) were infused over 45 minutes after HS. Different treatments were studied : RBC+plasma of rats or RBC+HEA. Four groups were studied : control (without HS), HS (without treatment), HS+RBC+rat plasma and HS+RBC+HEA. We evaluated survival to 300 min and hemodynamic parameters. eNOS, P-eNOS, Bax and Bcl2 levels in the aorta tissue were determined by Western Blot.

## Results

Plasma and HEA treatment improved significantly survival up to 300min and restored hemodynamics parameters (arterial pressure, heart rate, carotid blood flow). These results are in agreement with P-eNOS/eNOS ratio. The protein level showed that HS increased apoptosis in aorta as determined by Bax/Bcl2 ratio expression (*p* < 0.05). Plasma infusion resulted in significantly less apoptosis than HEA resuscitation. These results suggest that plasma may have a protective effect on vascular function.

## Conclusions

Plasma and HEA resuscitation have the same beneficial effects on survival and haemodynamic parameters. However, plasma seems to have additional benefical effect on apoptosis as compared to HEA, but the clinical significance remains to be determined.

HS: Hemorrhagic Shock, Voluven®: HEA, RBC: Red Blood Cells

